# The Characters of Diseased Animal Food

**Published:** 1855

**Authors:** 


### THE CHARACTERS OF DISEASED ANIMAL FOOD.

On this subject the Imperial and Central Society of Veterinary Medicine of France some time ago proposed the following questions:—

"1. Is it possible to ascertain by examination of butchers' meat (beef, veal, mutton, and pork), whether the animal was perfectly healthy when slaughtered.

"2. Are there any peculiar characters enabling one to determine whether butchers' meat, the animal either being entire, quartered, or otherwise divided, from atmospheric or other influences, or from an animal which has died from has been obtained from a healthy animal, but whose flesh has undergone changes fatigue, accident, want of care, privation of food, &c. Is such meat to be regarded as wholesome or unwholesome? In the latter case, what mischief is likely to be produced by its temporary or habitual use?

"Is it possible, from the inspection of an animal, either entire or divided, to determine whether, before dying or being slaughtered, it had been the subject, for a greater or less period of time, of such diseases as peripneumonia, phthisis, rot, measles, dropsy, &c.? If so, shew how the traces of these diseases may be recognised.

"4. Ought such meat to be consumed, or to be confiscated and destroyed?

"5. Are there any positive signs by which it may be determined from what animal any portion of butchers' meat has been taken?"

In answer to these questions, one essay only, by M. Soumille, of Avignon, was sent in. The following is an abstract of his replies:—

1. It is possible to ascertain whether an animal is healthy only when it is whole or quartered, except in diseases which manifest themselves in the entire system, such as measles, rot, cachexia, tubercular consumption, and *charbon*.

2. During rainy and misty seasons, meat, changed by atmospheric influences, remains soft; it dries with difficulty; it has a palish colour, and readily returns the impression of the finger. Such meat decomposes very rapidly, and contracts a putrid odour, which increases with time. M. Soumille has seen it even become black in two days. The meat can be cooked, but it is always soft and tasteless. In stormy seasons, the south winds exercise a pernicious influence on meat, especially lamb and veal. Beef and mutton escape; but they yield a broth deficient in nutritive materials. In winter, during severe frosts, meat is sometimes frozen; it then becomes very rigid; when cut with the knife, drops of coloured fluid exude from each fibre; it resists cooking, and does not cease to yield water. It ought not therefore to be consumed as food, being tasteless and indigestible. In the violent heat of summer, meat soon becomes black and decomposed more or less rapidly; the rapidity of the decomposition depends on the previous condition of the animals and on their food. In Avignon and the neighbourhood, fresh beef sometimes exhales an odour of onions; this is ascribed to the presence in the pastures of a plant of that kind.

The circumstances enabling us to determine whether an animal has died a natural death, are, when it is entire, the presence of disease of the viscera, coagulation of the blood in the vessels, accumulation of blood in the vessels of the great intestinal cavities, and sanguineous injection of the vessels of the cellular tissue. Separate portions of the meat are red, yield blood when cut into, and are of various colours on the surface. Nearly the same characters are observed, differing in intensity, in the flesh of animals which have been slaughtered after over-driving or deficient food. M. Soumille insists especially on the injection of the muscular flesh; but he does not consider that any real distinction can be drawn between a piece of meat taken from an animal which has been slaughtered after fatigue and from one which has died of disease. The former may be eaten; the latter ought to be rejected, although, from experiments on dogs, cats, ducks, and fowls, M. Soumille does not think it likely to produce mischief.

3. Examination of the entire animal, so that the viscera may be inspected, is the only means of ascertaining whether it has died of *charbon*, peripneumonia, or consumption. In sheep attacked with rot, examined when whole, the cellular tissue is infiltrated and riddled with small apertures. The flesh of sheep affected with dropsical cachexia is infiltrated with serous fluid, flabby, and colourless. Measles in pork is easily recognised by the presence of small, whitish granulations on the cut surface of the meat, especially in the lean portion: on exposure to the fire, a crackling sound is produced by the bursting of the little vesicles. In other respects,—as regards colour, smell, and consistency, measled meat has no peculiar characteristics. M. Soumille does not consider that it resists the process of cooking, that it yields a turbid, tasteless broth, or that it produces indigestion, diarrhœa, or other diseases. On the other hand, he admits that sausages made of it dry with difficulty, continue good for a shorter time, and soon become black and rancid, if not kept in a dry place.

4. M. Soumille would forbid the use of the meat of animals, whose leanness is coincident with disease, old age, and paleness of the flesh. He also proscribes, although persuaded of its harmlessness, the meat of animals that have been fatigued, or have not been sufficiently bled before being slaughtered, on account of its tendency to decomposition.

5. The last question received no rigorous answer.

*Gazette des Hôpitaux, October* 14, 1854.

[If an investigation of the means of detecting bad food is important in France, it is at least equally so in this country, the natives of which are proverbially more "beef-eaters" than their Gallic neighbours. As an instance of the amount of animal food which is consumed in this country, we may state that in 1853 there were sold in Smithfield 294,571 oxen, 1,518,040 sheep, 36,791 calves, and 29,593 pigs. The total sale of meat, dead and alive, for London consumption, is estimated at 483,388 oxen, 2,141,393 sheep, 132,976 calves, and 159,052 pigs.—EDITOR.]

